# Myocarditis and myasthenia gravis induced by immune checkpoint inhibitor in a patient with relapsed thymoma: A case report

**DOI:** 10.1002/ccr3.7039

**Published:** 2023-03-22

**Authors:** Peng Zhong, Cuizhen Zhang, Hongshan Guan, Jie Yan, Mengying He, Xiaoyang Zhou

**Affiliations:** ^1^ Department of Cardiology Renmin Hospital of Wuhan University Wuhan China

**Keywords:** case report, immune checkpoint inhibitors, immunosuppressants, myasthenia gravis (MG), myocarditis, steroids

## Abstract

Immune checkpoint inhibitors (ICIs)‐targeting CTLA4 and PD1 constitute a promising class of cancer treatment but are associated with several immune‐related adverse events (irAEs). A 55‐year‐old male patient with relapse thymoma was subjected to ICI therapy (PD‐1 antibody), 2 weeks later, the patient started to manifest including droopy eyelids, weak neck, arms, and legs, and shortness of breath. Then the patient was admitted to the hospital because of the MG symptoms. Arterial blood gases (ABGs) revealed the presence of hypercapnia. Noninvasive ventilation was utilized for respiratory support. At admission, increased serum troponin levels, coupled with interventricular conduction abnormalities were observed. On the second day after admission, the patient developed transient loss of consciousness and twitching of the muscles, and electrocardiography monitoring showed intermittent third‐degree atrioventricular block and ventricular pause necessitating temporary cardiac pacing. After excluding the possibility of acute coronary syndrome, intravenous steroids, intravenous immunoglobulin, pyridostigmine, and mycophenolate mofetil were sequentially initiated. 2 weeks later after treatment initiation, cardiac biomarkers and conduction abnormalities were recovered. 7 weeks later, MG symptoms were markedly improved. ICI‐related MG and myocarditis can be life‐threatening without appropriate management and clinicians should have a high index of suspicion for these irAEs in cancer patients receiving ICIs therapy. Steroids remain the cornerstone in the current management of irAEs due to the fast onset of action and high efficacy. However, in severe and refractory cases where no improvement is achieved despite high‐dose steroids, alternative immunosuppressants should be considered.

## Graphical abstract

Treatment with immune checkpoint inhibitors (ICIs) is often accompanied by immune‐related adverse events. ICIs‐induced myocarditis as manifested by serious conduction block can be life‐threatening without appropriate management. Careful monitoring and timely treatment are required when clinicians use ICIs for cancer treatment.

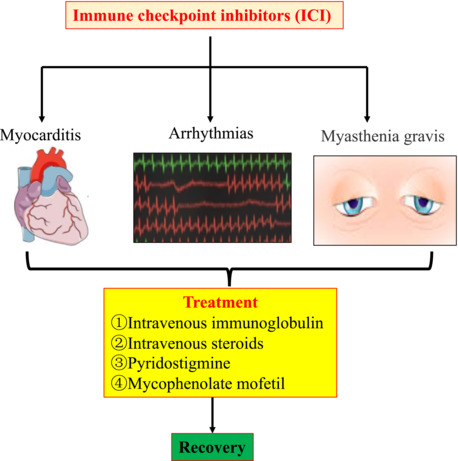

## 1 INTRODUCTION

Unbalancing the immune system with monoclonal antibodies targeting immune checkpoint receptors (ICIs) in cancer patients can generate dysimmune toxicities, called immune‐related adverse events (irAEs), that can potentially affect any tissue. Herein, we present a case of a 55‐year‐old man who developed ICI‐related myasthenia gravis (MG) and myocarditis following treatment of relapse thymoma with a PD‐1 inhibitor, toripalimab. To the best of our knowledge, few cases have been reported the serious conduction block coupled with myocardial injury as the main manifestation of myocarditis. This case report was written under the CARE guideline for case reports.^1^

## 2 CASE PRESENTATION

A 55‐year‐old male patient with relapse thymoma was subjected to ICI therapy (PD‐1 antibody). 12 days later, the patient started to manifest the symptoms of myasthenia gravis (MG) including droopy eyelids, weak neck, arms, and legs, and shortness of breath. Then the patient was admitted to the hospital because of symptoms of MG. On admission, physical examination showed oculomotor dysfunction, 3/5 strength of the neck musculature, and 4/5 strength in the upper and lower extremities with Babinski negative. No sensory impairment was noticed. The findings of the Head CT and MRI were normal. Due to the breathing difficulty and hypercapnia, noninvasive positive pressure ventilation (NPPV) was utilized for respiratory support and arterial blood gases (ABGs) were monitored to assess respiratory function (Figure 3A). Autoantibodies against acetylcholine receptors were screened and could not be detected serologically.

**FIGURE 1:**
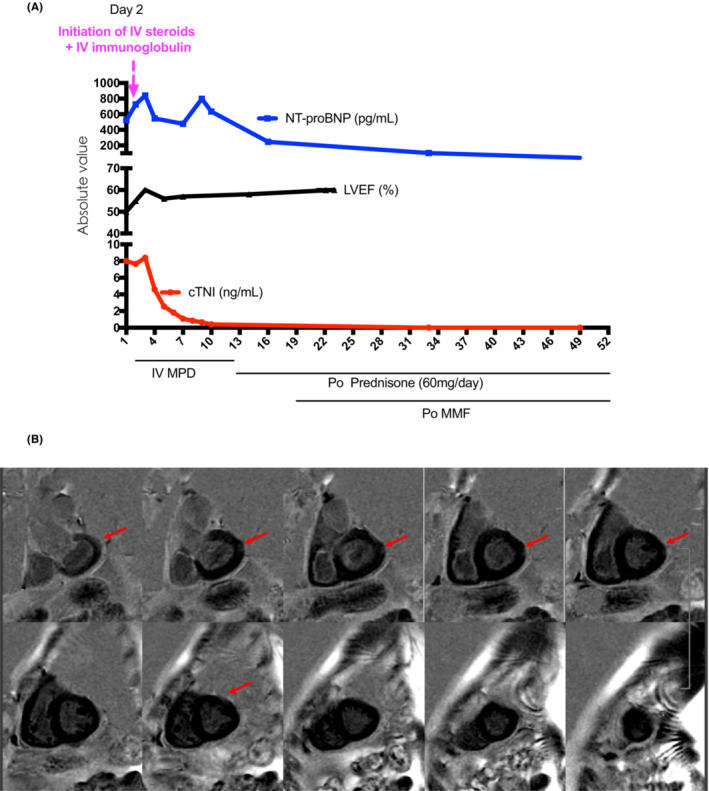
(A) The dynamic changes in cardiac troponin level, NT‐proBNP level, and LVEF in the patient with ICI‐related myocarditis during the hospital stay. (B) Cardiac MRI showed a possible late gadolinium enhancement (LGE) in the epicardial layer of the left lateral ventricular wall. MRI: magnetic resonance imaging. IV MPD: intravenous administration of methylprednisolone. Po: oral administration. MMF: mycophenolate mofetil. LVEF: left ventricular ejection fraction.

**FIGURE 2:**
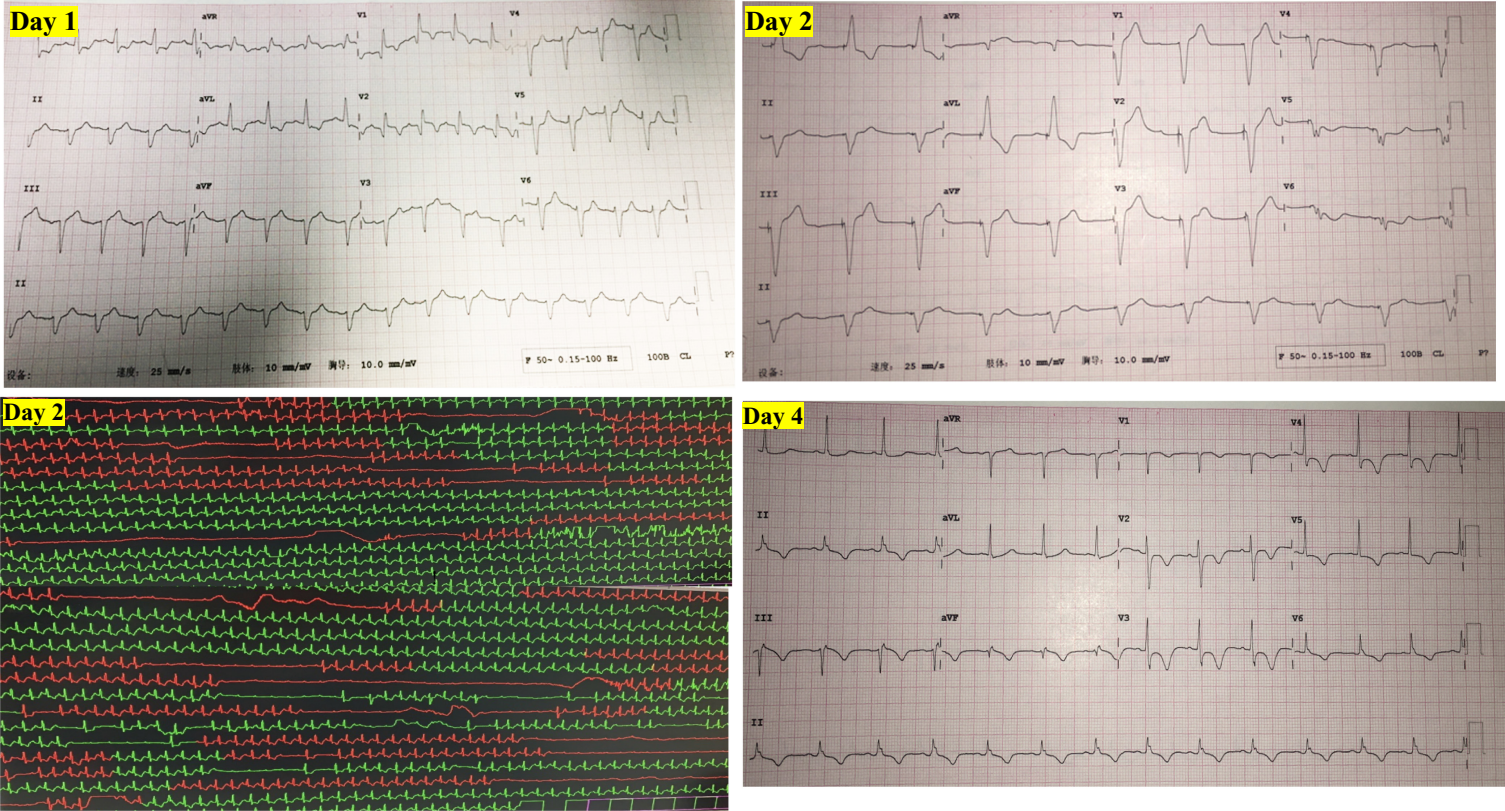
Dynamic changes of ECG of the patients in the initial 4 days after patient admission. When the patient was admitted, the ECG of Day 1 showed the presence of completely right bundle branch block morphology. Electrocardiography monitoring on Day 2 showed paroxysmal third‐degree atrioventricular block (IIIAVB) and ventricular pause with the longest pause lasting for 17 s. Then temporary cardiac pacing (VVI model, at a rate of 55 bpm) via the percutaneous femoral vein approach was initiated and intermittent pacing was documented by ECG on day 2. On Day 4, ECG showed the complete resolution of right bundle branch block morphology after steroid treatment.

On admission, an elevated serum level of troponins was also detected and 12‐lead ECG showed sinus rhythm with a completely right bundle branch block (CRBBB) (Note: electrocardiography obtained 1 month previously has documented normal ventricular conduction). The acute coronary syndrome was suspected and coronary computed tomography angiography (CTA) was performed and no apparent stenosis of the coronary arteries was identified. On Day 2, the patient developed transient loss of consciousness and twitching of the muscles, and electrocardiography monitoring showed paroxysmal third‐degree atrioventricular block (IIIAVB) and ventricular pause with the longest pause lasting for 17 s (Figure 2). Then temporary transvenous pacing was initiated and intermittent pacing could be documented by ECG (Figure 2). Serum troponins, NT‐proBNP levels, and echocardiography were monitored to evaluate the progression of cardiac injury and dysfunction (Figure 1A).

Based on the patient's clinical presentation and the above investigation, the diagnosis of irAEs was suspected and intravenous methylprednisolone (MPD, 1000 mg daily followed by a taper at half of the previous dose every 4 days) and intravenous immunoglobulin (IVIG, 20 g every 4 weeks) were initiated. 3 days later, ECG showed that CRBBB was fully resolved, coupled with a significant reduction in serum troponin level (Figure 2). On Day 12, 24 h of Holter monitoring showed less than 1% beats conferred by the temporary pacemaker, then the temporary pacemaker was removed. On Day 31, late gadolinium enhancement (LGE) of cardiac magnetic resonance imaging (CMR) identified a mild epicardial LGE in the lateral wall of the left ventricle (Figure 1B).

On Day 6, although intravenous use of steroids for 5 days, the patient still manifested dyspnea with hypercapnia, then pyridostigmine was added to improve muscle strength. On Day 16, NPPV was only supported intermittently as the symptom of dyspnea was partially resolved. On Day 18, due to no apparent improvement in the eyelid position and eye movements, additional immunosuppressive therapy with mycophenolate mofetil (MMF) was initiated. 5 days later, after the addition of MMF treatment on Day 23, the patient showed improved eyelid position and eye movements, coupled with an improved symptom of dyspnea at daytime, then NPPV was only performed at night, and the patient was encouraged to do breathing exercise at daytime. On Day 32, NPPV was suspended as the patient can tolerate it without NPPV.

**FIGURE 3:**
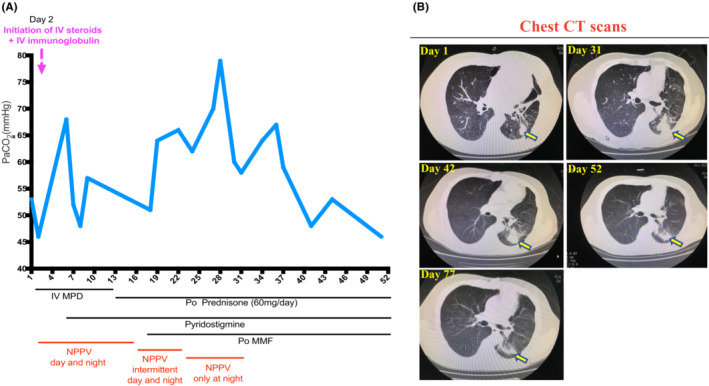
(A) The dynamic changes in arterial carbon dioxide pressure (PaCO2) of the patient during the hospital stay in response to treatment with intravenous methylprednisolone (IV MPD), intravenous immunoglobulin (IVIG), pyridostigmine, and mycophenolate mofetil (MMF), as well as respiratory support with noninvasive positive pressure ventilation (NPPV). (B) The dynamic changes of lung infection as revealed by repeated chest CT scans after treatment with antibiotics.

On Day 53, the patient's condition was stable and allowed to be discharged on oral prednisone, MMF, and ceclor. After discharge, the patient was allowed to reduce the oral dose of prednisolone by 10 mg every week, and MMF was continued for another week after the cessation of oral steroids. Ceclor was only used for 2 weeks and then suspended. At the follow‐up visit (25 days later after discharge), cardiac assessment including cardiac biomarkers (troponin, NT‐proBNP), ECG, and Echocardiogram were performed and showed no abnormal findings. The chest CT scan showed that the previous lung infection was gradually dissolved (Figure 3B). Muscle strength and physical performance return to normal.

## 3 DISCUSSION

There are increasing reports of autoimmune toxicity following ICI treatment. ICI can break up immunological homeostasis and reduce T‐cell tolerance. Therefore, inhibition of immune checkpoints can lead to the activation of autoreactive T‐cells, resulting in various irAEs similar to autoimmune diseases. Treatment with systemic steroids is the principal strategy against irAEs. The use of immune‐modulatory agents should be considered in cases of no response to steroid therapy. Here we reported the occurrence of ICI‐related MG and myocarditis in a patient with relapse thymoma who was successfully treated with steroids and immunosuppressive therapy coupled with supportive treatment.

In the present case, at about 19 days after ICI therapy, this patient presented with an increased cardiac biomarker and seriously advanced conduction block needing support with a temporary cardiac pacemaker. Fortunately, after a series of intravenous steroids and IVIG treatment, cardiac biomarkers, and conduction abnormalities were quickly resolved. ICI‐related myocarditis has a reported incidence of 0.04%–1.14% and it has a significantly higher associated mortality of 25%–50%.^2^ The clinical presentation of myocarditis can vary from asymptomatic elevations in cardiac biomarkers to severe decompensation with cardiogenic shock, which may be accompanied by serious arrhythmias, such as advanced atrioventricular block or ventricular tachycardia.^3^, ^4^, ^5^ The exact mechanism of ICI‐related myocarditis is unclear, but several lines of experimental evidence suggest that PD‐1 is protective against autoimmune‐related cardiac damage.^6^ The treatment of ICI‐associated myocarditis has largely been based on the use of glucocorticoids. For ICI‐related myocarditis, most case reports and case series track the response to steroids by monitoring troponin levels. If the troponin level begins to increase again, steroid dosing is increased and tapered over a longer period. If there is a lack of adequate clinical or biomarker response to steroids, consideration may be given to adding other immune modulators such as intravenous immunoglobulin (IVIG), mycophenolate, infliximab, and abatacept.^2^ Therefore, close cardiac monitoring and timely diagnosis and treatment of myocarditis in the setting of ICI therapy are important to reduce the fatal complications of the heart.

MG is a rare but life‐threatening complication of ICI‐related neurotoxicity, and it generally occurs within a month of drug use.^7^ The mortality rate of ICI‐related MG (irMG) was reported to be 29.8%–30.4% and respiratory paralysis is the major cause of death in irMG.^8^, ^9^ Therefore, timely diagnostic and therapeutic planning is necessary, particularly for patients with higher mortality risk. In the present case, after 2 weeks of ICI therapy, the patients quickly developed severe muscle weakness with respiratory dysfunction requiring ventilation support. Initial combination treatment with IV methylprednisolone and pulsed IVIG for as long as 17 days seems not to alleviate the MG symptoms in this patient. However, when adding the immunosuppressant agent (MMF) for about 5 days, a remarkable improvement in MG symptoms was observed in terms of eyelid position and eye movements as well as the reduced requirement of continuous NPPV support. Based on published cases relating to the treatment of irMG, the therapeutic strategies varied widely.^9^ There are plans to start with high‐dose methylprednisolone or IVIG, to start plasma exchange and IVIG initially regardless of clinical severity, and, alternatively, to use immunosuppressants like mycophenolate, methotrexate, cyclophosphamide, rituximab, natalizumab, bortezomib, and even tacrolimus for refractory cases.^9^ The experience of this patient suggests that initial aggressive treatment in terms of triple therapy may be needed for the successful treatment of irMG.

## 4 CONCLUSION

In summary, clinicians using ICIs should have a high index of suspicion for ICI‐related irAEs since they can be life‐threatening without appropriate management. Steroids remain the cornerstone in the current management of irAEs due to the fast onset of action and high efficacy. However, in severe and refractory cases where no improvement is achieved despite high‐dose steroids, alternative immunosuppressants should be considered.

## AUTHOR CONTRIBUTIONS

**peng zhong:** Conceptualization; visualization; writing – original draft. **Cuizhen Zhang:** Conceptualization; investigation. **Hongshan Guan:** Investigation. **Jie Yan:** Investigation. **Mengying He:** Investigation. **Xiaoyang Zhou:** Conceptualization; writing – review and editing.

## FUNDING INFORMATION

This work was supported by the National Natural Science Foundation of China (grant no. 81970331).

## CONFLICT OF INTEREST STATEMENT

The authors declare that they have no competing interests.

## ETHICAL APPROVAL

Ethical approval for case reports is waived by the institutional review board in the Renmin Hospital of Wuhan University. Consent to participate is not applicable.

## CONSENT

Written informed consent was obtained from the patient for publication of this case report and any accompanying images. A copy of the written informed consent is available for review by the Editor‐in‐chief of this journal.

## Data Availability

Not applicable.
